# On the role of the MAGUK proteins encoded by *Drosophila varicose *during embryonic and postembryonic development

**DOI:** 10.1186/1471-213X-8-55

**Published:** 2008-05-18

**Authors:** André Bachmann, Margarete Draga, Ferdi Grawe, Elisabeth Knust

**Affiliations:** 1 Institut für Genetik, Heinrich-Heine-Universität Düsseldorf, Universitätsstr. 1, 40225 Düsseldorf, Germany; 2 Max-Planck-Institute of Molecular Cell Biology and Genetics, Pfotenhauerstr. 108, 01307 Dresden, Germany; 3 Division of Cell and Developmental Biology, College of Life Sciences, JBC/MSI/WTB Complex, University of Dundee, Dow Street, Dundee, DD1 5EH, Scotland, UK

## Abstract

**Background:**

Membrane-associated guanylate kinases (MAGUKs) form a family of scaffolding proteins, which are often associated with cellular junctions, such as the vertebrate tight junction, the *Drosophila *septate junction or the neuromuscular junction. Their capacity to serve as platforms for organising larger protein assemblies results from the presence of several protein-protein interaction domains. They often appear in different variants suggesting that they also mediate dynamic changes in the composition of the complexes.

**Results:**

Here we show by electron microscopic analysis that *Drosophila *embryos lacking *varicose *function fail to develop septate junctions in the tracheae and the epidermis. In the embryo and in imaginal discs *varicose *expresses two protein isoforms, which belong to the MAGUK family. The two isoforms can be distinguished by the presence or absence of two L27 domains and are differentially affected in different *varicose *alleles. While the short isoform is essential for viability, the long isoform seems to have a supportive function. Varicose proteins co-localise with Neurexin IV in pleated septate junctions and are necessary, but not sufficient for its recruitment. The two proteins interact in vitro by the PDZ domain of Varicose and the four C-terminal amino acids of Neurexin IV. Postembryonic reduction of *varicose *function by expressing double-stranded RNA affects pattern formation and morphogenesis of the wing and the development of normal-shaped and -sized eyes.

**Conclusion:**

Expression of two Varicose isoforms in embryonic epithelia and imaginal discs suggests that the composition of Varicose-mediated protein scaffolds at septate junctions is dynamic, which may have important implications for the modulation of their function.

## Background

Membrane-associated guanylate kinases (MAGUKs) form a family of scaffolding proteins, engaged in the organisation of multiprotein complexes, which are often associated with cellular junctions or signalling complexes, such as the vertebrate tight junction (TJ) or the *Drosophila *septate junction (SJ) in epithelial cells or the neuromuscular junction (NMJ). Their capacity to serve as a platform for recruiting larger protein assemblies results from the presence of several protein-protein interaction domains: one to three PDZ (PSD-95/Discs large/zonula occludens (ZO)-1)-domains, an SH3-(Src homology-3)-domain and a guanylate kinase (GUK)-domain. Some members additionally contain one or two L27 (Lin-2/Lin-7)-domains in their N-terminus [1,2]. This modular structure is ideally suited to recruit a variety of components into a protein complex, the composition of which often depends on the cell type and/or developmental stage. In addition, MAGUK-encoding genes often give rise to more than one isoform by alternative splicing, thus increasing the possibility of multiple interactions, localisations and/or functions. For example, the vertebrate membrane-associated palmitoylated protein 4 (MPP4) encodes a retina-specific isoform, which lacks the L27 domain in its N-terminus [3]. Similarly, *Drosophila discs large *(*dlg*) expresses an isoform (DlgA) lacking the L27 domain, which is unable to bind to *D*Lin-7 in epithelia, whereas an isoform containing the L27 domain (Dlg-S97) can associate with *D*Lin-7 in the neuromuscular junction [4,5]. *Drosophila *Polychaetoid, the orthologue of mammalian ZO-1, encodes two isoforms, one of which is localised apically, whereas the other one distributes more broadly along the lateral membrane of epithelial cells of the wing imaginal disc [6]. Finally, *Drosophila stardust (sdt) *expresses a distinct isoform in the embryo, characterised by the presence of a particular exon, which is not expressed in the eye [7]. Taken together, MAGUK proteins provide a versatile platform for the dynamic assembly of protein complexes in various cell types.

Septate junctions (SJs) in epithelia of *Drosophila *reside in the lateral membrane, basal to the zonula adherens (ZA). Two kinds of SJs are known, pleated SJs and smooth SJs. Pleated SJs are present in most ectodermally derived epithelia of the embryo, i. e. the epidermis, the tracheae, the salivary glands, the fore- and the hindgut, but they are excluded from the amnioserosa and the Malpighian tubules. They are characterised by ladder-like septa spanning the space between the lateral membranes of adjacent cells. They are first visible in these epithelia around stage 14 and become further elaborated by the end of embryogenesis [8,9]. SJs can be found in epithelia of arthropods [10] and at the blood-brain barrier of both arthropods and chordates [11] [reviewed in [12]]. They serve as barriers between different compartments by providing a paracellular diffusion barrier, a function equivalent to that of vertebrate tight junctions. In addition, SJs are involved in the control of tracheal tube size [13]. The molecular components in *Drosophila *septate junctions include transmembrane proteins, such as Neurexin IV (NrxIV) [14], Neuroglian, Gliotactin and a Na^+^/K^+ ^ATPase [15,16], the cell surface protein Lachesin [17] as well as scaffolding proteins, such as the FERM (4.1/ezrin/radixin/moesin) family member Coracle (Cora) [18] and the MAGUK Discs large (Dlg) [19]. Some of them are orthologues to vertebrate TJ proteins, such as Megatrachea or Sinuous, two claudin-like proteins [10,20], or contactin [21] [reviewed in [12,22]].

Recently it has been shown that septate junctions contain an additional member of the MAGUK protein family, encoded by *varicose*, which is required for proper localisation of other septate junction components and tracheal tube size development [13]. Here we show by electron microscopy that *varicose *mutant embryos fail to develop proper pleated SJs. We further demonstrate that two different transcripts and proteins are expressed in the embryo, which are differentially affected by different *varicose *alleles, pointing to individual functions. In addition, we provide data to show that *varicose *is expressed in the wing and eye imaginal discs and necessary for their normal development.

## Results

### *varicose *expresses two MAGUK isoforms in the embryo

**Figure 1:**
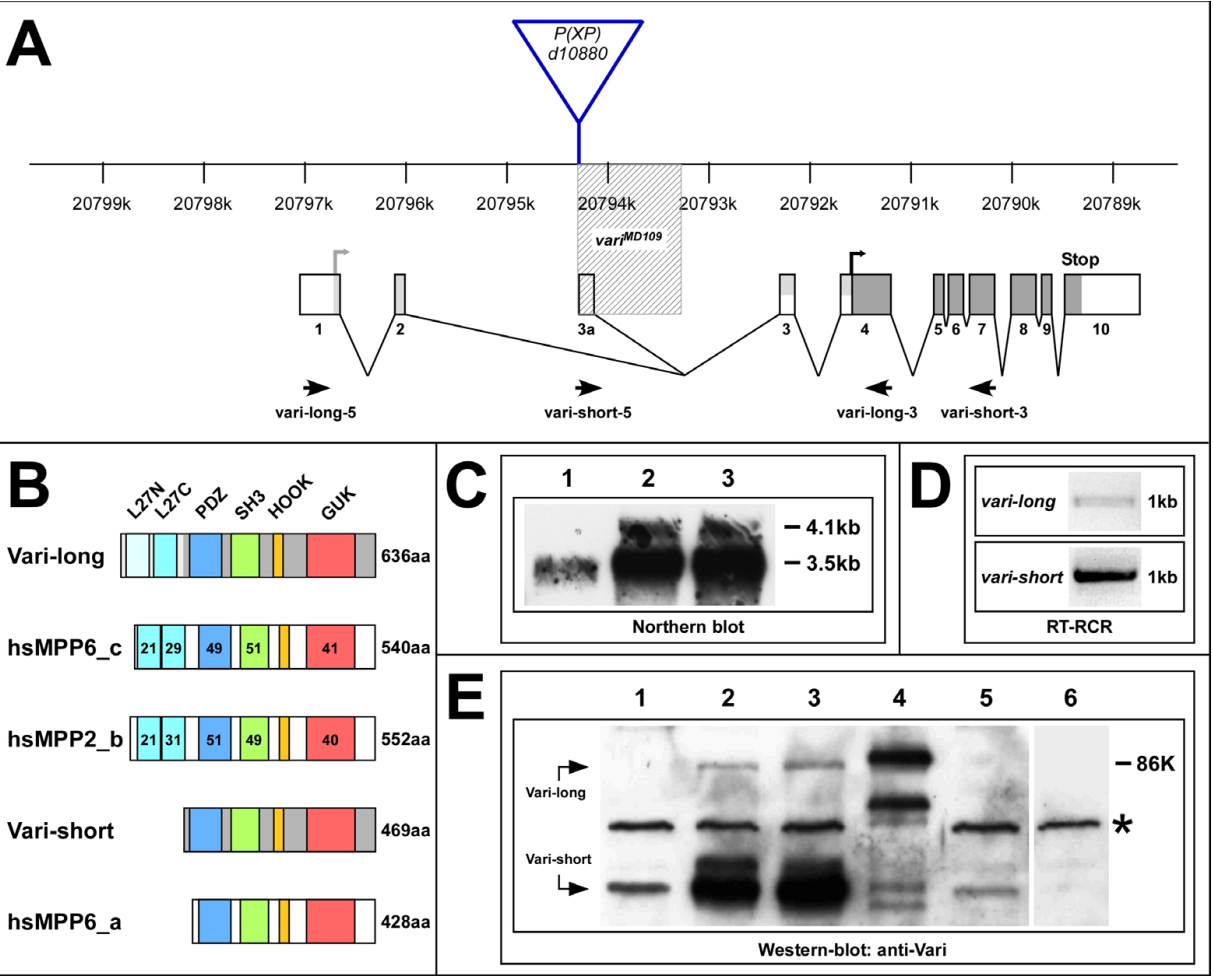
***varicose *encodes a MAGUK protein**. (A) Exon-intron structure of the *vari *locus (scale bar according to FlyBase). Two different transcripts could be isolated: the *vari-long *transcript contains exons 1, 2, 3 to 10 with the translational start residing in exon 1; *vari-short *consists of exons 3a, 3 to 10 with the translational start site in exon 4 (dark grey boxes: exons common to both transcripts, light grey boxes: exons specific to *vari-long*, white boxes: UTR). Mobilisation of the *P(XP)*-element *d*10880 yielded the *vari-short*-specific mutant allele *vari*^*MD*109^, which removes exon 3a and adjacent 3'-intronic DNA (shaded box). Primer pairs *vari-long-5/3 *and *vari-short-5/3 *were used to detect the corresponding *vari *transcripts in the wild-type (D) and different *vari *alleles (see Fig. 5). (B) Structure and size comparison of the MAGUK proteins Vari-long and Vari-short with their human homologs hsMPP6_c (GenBank accession number EAW93810), hsMPP6_a (GenBank accession number EAW93808) and hsMPP2_b (GenBank accession number EAW51656), respectively. The percentages of amino acid identities of the domains with respect to the corresponding domains of Vari-long are shown. (C) Northern blot of 5 μg poly(A^+^) RNA from staged wild-type embryos (1 = 0–4 h, 2 = 4–12 h, 3 = 12–24 h) hybridised with a probe that detects both *vari-long *and *vari-short *transcripts. (D) RT-PCR on total RNA from wild-type embryos (> 8 h old) with primer pairs *vari-long-5/3 *and *vari-short-5/3 *detects single *vari-long*- and *vari-short*-transcripts. (E) Western blot analysis of protein lysates from staged wild-type embryos and embryos of different genotypes (> 8 h old), probed with an anti-Vari antibody that recognises both Vari-long and Vari-short (1 = wt, 0–4 h, 2 = wt, 4–12 h, 3 = wt, 12–24 h, 4 = *daG32>UAS Flag-vari-long*, 5 = *daG32>UAS vari-RNAi*, 6 = *Df(2L)DS6*). Overexpressed Flag-Vari-long in lane 4 is slightly bigger than endogenous Vari-long due to the N-terminal Flag-tag. The protein amount loaded per lane equals 5 embryos with the exception of lysates from *daG32>UAS Flag-vari-long *embryos (equals 0.5 embryos). The lysate from *Df(2L)DS6 *embryos serves as a negative control and an unspecific, cross-reacting band (asterisk) as a loading control.

In *Drosophila *epithelia, the MAGUK-proteins Stardust (Sdt) and Discs Large (Dlg) are required to organise protein complexes in the subapical and the lateral membrane, respectively. Sdt recruits the transmembrane protein Crumbs (Crb) and two other scaffolding proteins, *D*Lin-7 and *D*PATJ, into a complex, which is localised subapically, i. e. in a small region of the apical plasma membrane just apical to the *zonula adherens*, in epithelial and photoreceptor cells [reviewed in [23]]. We were interested in identifying additional partners of the Crumbs complex, which might have a function in the organisation of epithelia of the *Drosophila *embryo. Therefore, we took advantage of the recently published protein interaction map [24] to screen for partners of *D*Lin-7, a component of the Crumbs complex [5,25]. One of the putative partners identified in this screen is encoded by *Drosophila *CG9326, located at 38E10 on the left arm of the second chromosome. It is predicted to encode three isoforms, which differ in their size and are the result of differential transcriptional initiation and alternative splicing (FlyBase) (Fig. 1A). As demonstrated recently [13], these proteins are encoded by *varicose *(*vari*; see below) and define a new subgroup of MAGUK proteins, which includes the mammalian proteins MPP2 and MPP6/VAM-1/Pals2 (Fig. 1B). Previously, only a single L27 domain was predicted in the longer isoform. When using SMART and reciprocal BLASTs (see Materials and Methods), two L27 domains are predicted. This is in agreement with the domain structure of orthologues from the honeybee, mouse, *Xenopus *and zebrafish (see Additional file 1). Strikingly, human MPP6 also encodes two isoforms of different size, MPP6_a and MPP6_c, the longer of which carries an extended N-terminal region with two L27 domains.

The expression of two different transcripts in the embryo was verified by northern blots and RT-PCR. Using a probe common to all predicted transcripts, two mRNAs of about 3.5 and 4.1 kb were detected on northern blots with poly(A^+^)-RNA of staged wild-type embryos, with the shorter one being much more abundant than the longer one (Fig. 1C). The expression of the two different transcripts was confirmed by RT-PCR, using transcript-specific primers (Fig. 1D). However, RT-PCR experiments failed to detect *vari *transcripts encoding the predicted isoform D (Flybase), suggesting that only isoforms B and C are expressed in the developing embryo, which represent the short and the long Vari isoform, respectively. In-situ hybridisations with a probe common to both transcripts reveal that *vari *is expressed in most ectodermally derived embryonic epithelia, i. e. the epidermis, the fore- and the hindgut, the tracheae and the salivary glands, but not in the Malpighian tubules (data not shown).

Western blot analysis with antibodies raised against a recombinant protein containing the PDZ- and the SH3-domain (anti-Vari) revealed that two variants of ~60 and ~80 kDa are present in extracts of wild-type or *vari*-hemizygous embryos (Fig. 1E). The smaller isoform, which matches the predicted size (53 kD), was already detectable in extracts from 0–4 hour embryos, but was much more abundant during later stages of embryogenesis. The larger isoform (predicted size 72 kD) was only detectable in embryos older than 4 hours, and present in only very small amounts compared to the shorter form. Western blots from embryos expressing a Flag-tagged Vari-long protein confirmed the specificity of the larger isoform (Fig. 1E, lane 4). Both proteins were almost completely or completely absent in extracts of embryos expressing *vari-RNAi *or embryos homozygous for a deficiency, which removes the entire gene, respectively (Fig. 1E, lanes 5 and 6).

**Figure 2:**
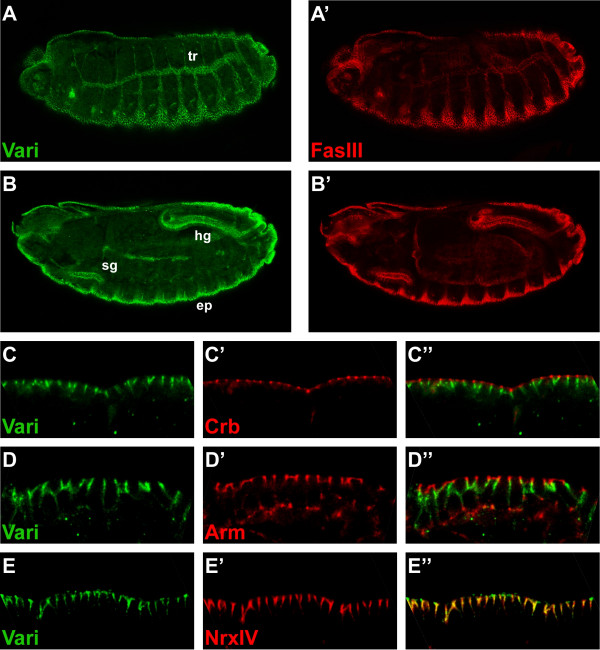
**Varicose localises to septate junctions in the embryo**. (A-B) A wild-type embryo of stage 15, in different focal planes, stained with anti-Vari (green) and anti-Fasciclin III (red). Vari is expressed in many epithelia of ectodermal origin. sg: salivary glands, tr: tracheae, ep: epidermis, hg: hindgut. (C-E) Epidermis of stage 15 wild-type embryos, stained with anti-Vari (green, C-E), anti-Crb (C'), anti-Arm (D'), and anti-NrxIV (E') (red), respectively. C"-E" show the merged images. Vari co-localises with the septate junction component NrxIV, basal to Crb and Arm. In A-B anterior is left and dorsal is up. In C-E apical is up.

Similar as shown recently [13], our antibody recognised an epitope in most ectodermally derived epithelia of wild-type embryos, i. e. in the epidermis, the tracheae, the salivary glands, the fore- and the hindgut, but not in the Malpighian tubules (Fig. 2A,B). In these tissues, the protein detected by the antibody localised basal to the ZA, as revealed by double-staining with anti-Crumbs (Crb) and anti-Armadillo (Arm), respectively (Fig. 2C,D). Vari co-localised with the septate junction components Neurexin IV (NrxIV) and Discs large (Dlg) (Fig. 2E and data not shown), confirming its association with septate junctions.

### *varicose *is required for septate junction development

We induced a *vari *mutation by imprecise excision of a P-element of the line *P(XP)d10880 *(Exelixis collection at Harvard), which localised immediately 5' to the transcription start site of the smaller transcript (Fig. 1A). One out of 17 homozygous lethal excision lines isolated failed to complement deficiency *Df(2L)DS6 *and *vari *alleles *vari*^03953*b*^, *vari*^327^*, vari*^*R*979^*, vari*^*R*3^* and vari*^*F*033 ^[26]. The mutant excision line carries a deletion that removes 1.103 basepairs of genomic DNA, thus completely eliminating exon 3a, the first exon of the transcript encoding the shorter isoform (Vari-short) plus part of the following intron (Fig. 1A, shaded box). Consistent with this result, embryos homozygous mutant for this small deletion did not express any Vari-short protein (see below). The genetic and molecular data classified the mutation identified here as a *vari *allele, which is called *vari*^*MD*109 ^from here on.

**Figure 3:**
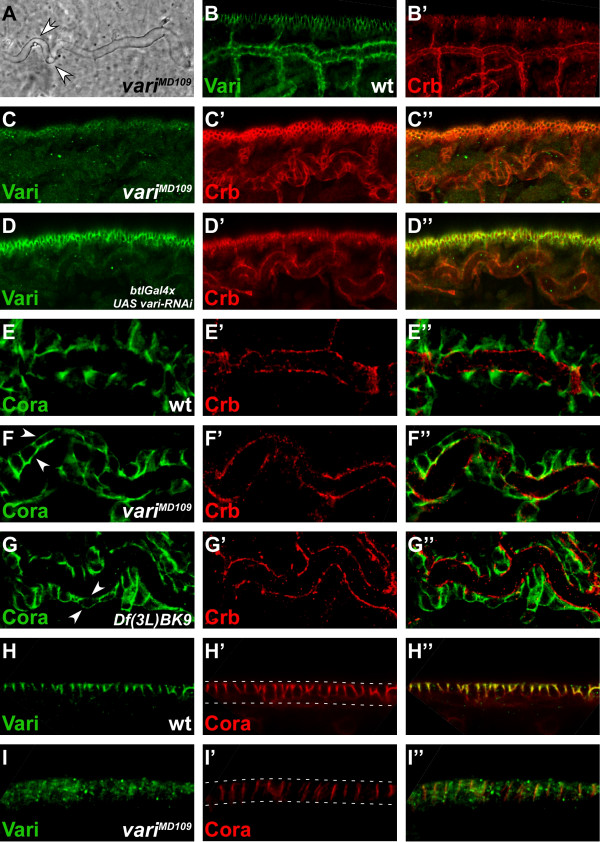
**Varicose is required for correct tracheal tube and epidermis formation**. (A) Cuticle preparations of *vari*^*MD*109 ^mutant embryos exhibit convoluted tracheae (white arrows). (B, B') Wild-type embryo of stage 15 stained with anti-Vari (green) and anti-Crb (red). Vari localises at the SJ, basal of Crb. Wild-type tracheae appear straight in contrast to the convoluted tracheae in A. (C) *vari*^*MD*109 ^mutant embryo of stage 15 stained with anti-Vari (green) and anti-Crb (red). Vari is lost from the tracheae and the epidermis, while apical Crb is not affected. Tracheae appear convoluted. (D) Stage 15 embryo with targeted knockdown of *vari *in the tracheae of embryos by using *btlGal4 *(*btlGal4>UAS vari-RNAi*), stained with anti-Vari (green) and anti-Crb (red). Vari is reduced to background levels in the tracheae, but not affected in the epidermis. Apical localisation of Crb is not affected in the tracheae. (E) Dorsal tracheal trunk of a wild-type embryo of stage 15, stained with anti-Coracle (Cora; green) and anti-Crb (red). Cora localises in the SJ, basal to Crb. (F) Dorsal tracheal trunk of a *vari*^*MD*109 ^mutant embryo of stage 15, stained with anti-Cora (green) and Crb (red). Cora is delocalised to apical and basal sites (white arrows), whereas Crb remains in its apical position. (G) Dorsal tracheal trunk of a *Df(3L)BK9 *mutant embryo of stage 15, in which the *NrxIV *locus is deleted, stained with anti-Cora (green) and anti-Crb (red). As in *vari*^*MD*109 ^mutant embryos, Cora becomes mislocalised to apical and basal positions (white arrows) in the absence of NrxIV, while apical localisation of Crb is not affected. (H) Epidermis of a wild-type embryo of stage 15, stained with anti-Vari (green) and anti-Cora (red). Both proteins are co-localised at the SJ. (I) *vari*^*MD*109 ^mutant embryo of stage 15, stained with anti-Vari (green) and anti-Cora (red). The amount of Cora is reduced and the remaining Cora protein is mislocalised along the whole lateral membrane. In B-D and H-I apical is up. White dotted lines in H' and I' mark the apical and basal side of the epithelial cells, respectively.

Embryos homozygous mutant for *vari*^*MD*109 ^or transheterozygous for *vari*^*MD*109^*/vari*^03953*b *^died at the end of embryogenesis with convoluted tracheal tubes (Fig. 3A), a phenotype similar to that described for *vari*^03953*b *^[13,26]. Apical-basal polarity was not affected, as revealed by proper apical localisation of Crb (Fig. 3B,C). The same phenotype was achieved by specifically knocking down *vari *function in the tracheae by expressing *vari-RNAi *by means of *btlGal4 *(Fig. 3D). Septate junction components, such as Coracle or Neurexin IV, which are restricted to the lateral membrane in wild-type embryos, became distributed to all membranes in *vari *mutant embryos, both in the tracheae (Fig. 3E,F) and the epidermis (Fig. 3H,I). This phenotype was reminiscent to that achieved in the absence of *Neurexin IV *function (Fig. 3G).

**Figure 4:**
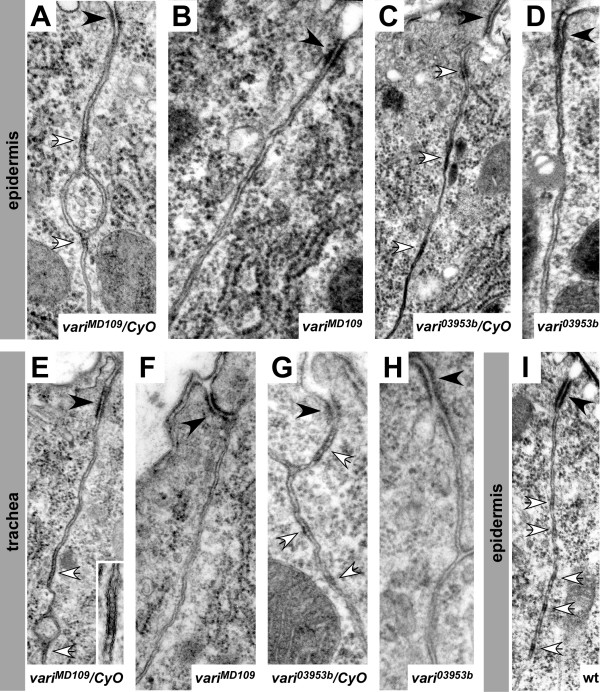
**EM analysis reveals defective septate junctions in *vari *mutants**. Epidermis (A-D, I) and tracheae (E-H) of stage 16 embryos. (A, E): *vari*^*MD*109^*/CyO-twi-GFP*; (B, F): *vari*^*MD*109^; (C, G): *vari*^03953*b*^*/CyO-twi-GFP*; (D, H): *vari*^03953*b*^; (I): wild-type. Adherens junctions (black arrowheads) and developing septate junctions (white arrows) can be distinguished in wild-type embryos. The inset in E shows the regularly aligned septa of pleated septate junctions between adjacent cells. Septate junctions are absent in homozygous *vari*^*MD*109 ^(B, F) and *vari*^03953*b *^(D, H) mutant embryos, while adherens junctions are well developed (black arrowheads).

Based on the failure to properly localise the septate-junction-associated proteins Neurexin IV and Coracle (Cora) in *vari *mutant embryos (Fig. 3 and [13]) Wu et al. suggested that these embryos fail to properly establish and/or maintain the septate junctions. In order to prove this, we carried out electron microscopic analysis of wild-type (or heterozygous) and *vari *mutant embryos. In wild-type embryos, pleated septate junctions are clearly distinguishable from stage 16 onwards [9] by the presence of septa, which span the space between the lateral membranes of neighbouring cells, both in the epidermis (Fig. 4A,C,I) and the tracheae (Fig. 4E,G). Homozygous mutant *vari*^*MD*109 ^embryos of the same stage lacked septate junctions in these tissues (Fig. 4B,F). Similarly, septate junctions were not detected in *vari*^03953*b *^homozygous mutant embryos (Fig. 4D,H). The ZA was not affected in epithelia of the mutant embryos and still formed a continuous apical belt. This result finally proves that *vari *has an essential function in the formation of the pleated septate junctions during embryonic development.

### *varicose *alleles differentially affect the two protein isoforms in the embryo

**Figure 5:**
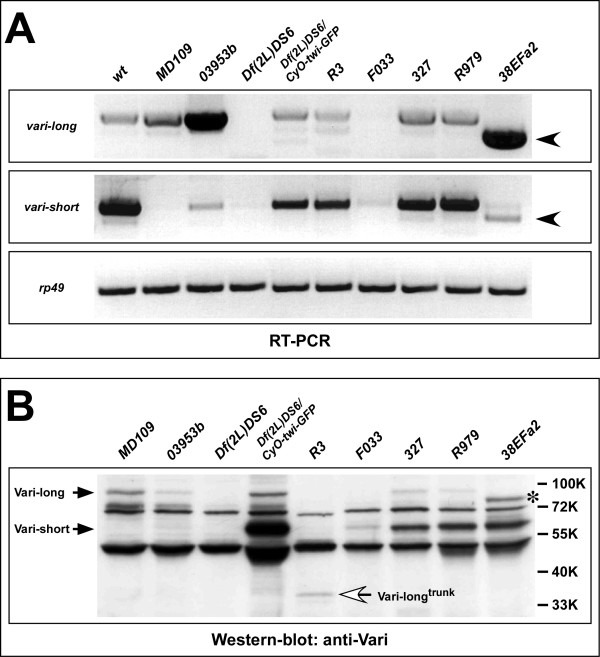
**RT-PCR- and western blot-analysis of *vari*-mutant alleles**. (A) RT-PCR on total RNA of wild-type and different *vari*-mutant embryos (> 8h), using *vari-long*- and *vari-short*-specific primer pairs *vari-long-5/3 *or *vari-short-5/3 *(see Fig. 1A). No *vari-long *transcripts can be detected in embryos homozygous for the deficiency *Df(2L)DS6 *and the allele *vari*^*F*033^. *vari-long *transcripts are slightly more abundant in *vari*^*MD*109 ^and strongly enriched in *vari*^03953*b *^embryos. Both *vari-long *and *vari-short *transcripts are truncated in *vari*^38*EFa*2 ^(black arrowheads). *vari-short *transcripts are absent in *vari*^*MD*109^, strongly reduced in *vari*^*F*033 ^and *vari*^03953*b*^, but only mildly affected in *vari*^*R*3^, *vari*^327 ^and *vari*^*R*979^. Transcripts from the *rp49 *gene serve as internal loading control. (B) Western blot analysis of the same mutant alleles as in A. Vari-long protein is present in wild-type amounts in *vari*^*MD*109^, absent in embryos homozygous for the deficiency *Df(2L)DS6 *and the allele *vari*^*F*033^, reduced in *vari*^03953*b*^,*vari*^327 ^and *vari*^*R*979 ^and truncated in *vari*^*R*3 ^(white arrow) and *vari*^38*EFa*2 ^(asterisk). Vari-short protein is absent in *vari*^*MD*109^, in the deficiency *Df(2L)DS6 *and in *vari*^*R*3 ^and reduced in the remaining alleles. RT-PCR and western-blot analysis of the deficiency *Df(2L)DS6*, which removes the *vari *locus, serves as negative control.

The molecular lesions in the DNA of several *vari *alleles, which exhibit a weak, an intermediate or a strong mutant phenotype, respectively, have been described [13]. In none of these mutant embryos, nor in embryos mutant for *vari*^*MD*109^, any localised Vari protein could be detected in the embryo (data not shown) [13]. We were interested to find out how the different alleles affect the expression of the two Vari isoforms and whether there is any relationship between the strength of the mutant phenotype of a given allele as determined by genetic analyses and the expression of the two Vari isoforms. Therefore, we performed RT-PCR and western blots on extracts of homozygous mutant embryos (Fig. 5). As expected, embryos mutant for the intermediate allele *vari*^*MD*109^, in which the transcription start site of the smaller form is deleted (Fig. 1A), did not express *vari-short *RNA or Vari-short protein, but still exhibited Vari-long protein expression, which seemed even slightly up-regulated. In the intermediate allele *vari*^03953*b*^, which carries a 17 bp deletion in the third common intron, very low amounts of *vari-short *RNA and protein were expressed. Although *vari-long *transcripts were upregulated in the mutant embryos, the amount of Vari-long protein was reduced in comparison to wild-type. The null allele *vari*^*R*3 ^carries a point mutation leading to a premature stop codon in the region encoding the Hook domain, which should result in a truncation of both protein isoforms. As expected, this mutation did not affect the size of the two transcripts. Western blots revealed that the long isoform was in fact truncated: a protein of about 40 kD, corresponding to a truncated Vari-long (calculated size: 38 kD) could be detected. The absence of any truncated short isoform suggested that this truncated form was not stable. The null allele *vari*^*F*033^, which carries a transposon in the intron between exons 3a and 3, did not express Vari-long and only very low amounts of Vari-short protein. In contrast, the insertion line *P(XP)d10880*, which carries a P-element in the intron between exon 2 and 3a (see Fig. 1A) expressed normal amounts of both proteins (data not shown). The mutation in *vari*^327^, also characterised as a null allele, alters the splice acceptor site of the common exon 6.

Therefore, one would expect that exon 6 is skipped. As a consequence, both transcripts should be reduced by 162 nucleotides and both proteins should lack 54 amino acids. Surprisingly, *vari*^327 ^mutant embryos still expressed wild-type sized transcripts and proteins. However, sequencing the RT-PCR product obtained by amplification with primers vari-short-5 and vari-short-3 (Fig. 1A) revealed, that the mutant splice site is skipped, and the next AG-site inside exon 6 is used as a splice acceptor instead. This results in a 15 bp deletion, which exactly removes 5 amino acids (IINVK) of the SH3-domain, without affecting the reading frame. This deletion is too small to be detected in the length of the RT-PCR product or the protein. In the strong allele *vari*^*R*979^, which carries a single point mutation, resulting in the exchange of an arginine to a cysteine in the Hook domain, the mutation did not have any effect on the size of the two proteins, but Vari-long seemed to be present in lower amount. In the weak allele *vari*^38*EFa*2^, which carries a mutation in the splice acceptor site of exon 3, the RNAs of both isoforms were reduced in length, but, as expected, only the long protein isoform was truncated. Taken together, the results obtained by western blot analysis showed that in most *vari *mutant alleles at least one of the protein isoforms is affected.

### Varicose is necessary, but not sufficient for Neurexin IV localisation

**Figure 6:**
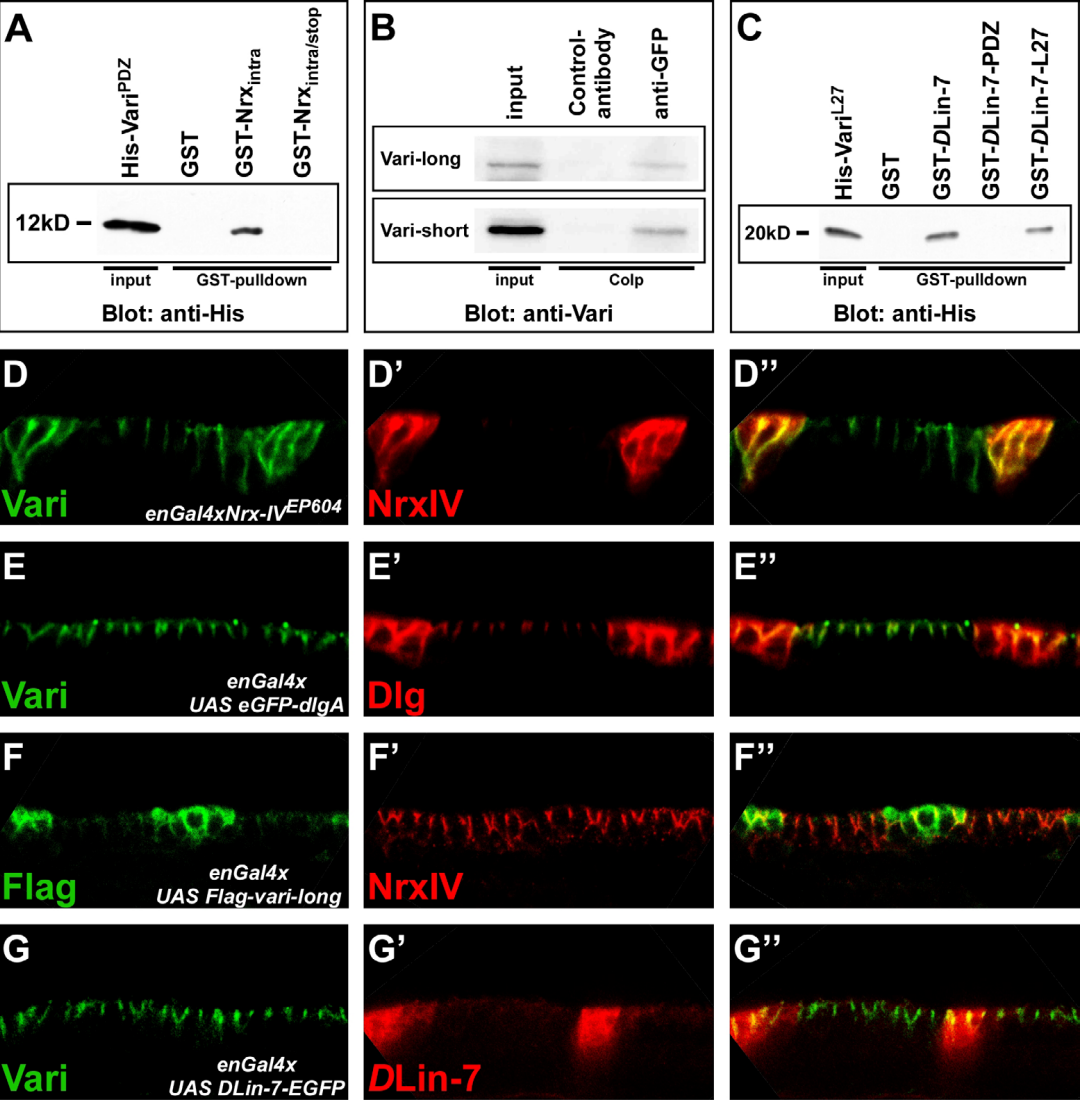
**Vari interacts with NrxIV in vitro and in vivo**. (A) A GST-NrxIV fusion protein comprising the entire intracellular domain of NrxIV (including the PDZ-binding motif-EIFI) pulls down a His-tagged Vari transgene that contains only the PDZ domain (Pro^169^-Val^270^) in vitro. However, a GST-NrxIV fusion protein including a C-terminally truncated intracellular domain of NrxIV (lacking the PDZ-binding motif-EIFI) does not. (B) Co-immunoprecipitation assay from embryonic protein extracts (> 8 h; genotype: *NrxIV-GFP/TM6B*) reveals that in the embryo NrxIV forms a complex with Vari-long and Vari-short. (C) Both GST-*D*Lin-7 and GST-*D*Lin-7-L27 fusion protein (containing only the L27 domain of *D*Lin-7), pull down the His-tagged Vari N-terminus (Ser^5^-Tyr^157^), which comprises the putative L27 domains, while GST-*D*Lin-7-PDZ fails to pull down Vari. (D-G) Wild-type embryos of stage 15 overexpressing different UAS-transgenes in a striped pattern using *enGal4*. Green: anti-Vari, Red: anti-NrxIV (D, F); Green: anti-Vari, Red: anti-Dlg (E); Green: anti-Vari, Red: anti-*D*Lin-7 (G). Recruitment of Vari by ectopic NrxIV is specific. In D-G apical is up.

The C-terminus of Neurexin IV was able to pull-down the recombinant, His-tagged PDZ-domain of Varicose when fused to GST (Fig. 6A) [13]. We could further identify the C-terminal PDZ-binding motif-EIFI of Neurexin IV as being crucial for this interaction, since its removal (GST-Nrx_intra/stop_) completely abolished the interaction (Fig. 6A). To confirm that these two proteins also form a complex in vivo, we made use of a protein trap line expressing a Neurexin IV-GFP fusion protein, in which the P-element is inserted in the first intron and GFP is fused in frame right after the signal sequence (N. Muschalik and E. Knust, unpublished). Immunoprecipitation of Neurexin IV-GFP with an anti-GFP antibody from embryonic extracts co-precipitated both Varicose isoforms (Fig. 6B). It has recently been shown that Neurexin IV is necessary to recruit Varicose to the septate junctions [13]. To demonstrate that it is also sufficient, we overexpressed Neurexin IV in the posterior compartment of each segment, making use of an EP-insertion in the *Neurexin IV *locus. This induced a moderate enrichment of Vari protein in regions of the cell with higher NrxIV protein levels (Fig. 6D). Overexpression of another SJ component, DlgA, failed to recruit Vari to ectopic sites (Fig. 6E), further demonstrating the specificity of the NrxIV-Vari interaction. In contrast, Vari-long did not recruit more NrxIV to the membrane (Fig. 6F), suggesting that Vari is necessary, but not sufficient for NrxIV localisation.

Varicose has initially been described as an interaction partner of *D*Lin-7 in a yeast two-hybrid screen [24]. This interaction could be confirmed by pull-down experiments. A GST-fusion protein, containing the L27-domain of *D*Lin-7, pulled down the recombinant L27 domains of Vari-long, fused to a His-tag. The PDZ-domain of *D*Lin-7 was unable to mediate this interaction (Fig. 6C). However, unlike NrxIV, overexpression of *D*Lin-7 in the embryonic epidermis had no effect on the distribution of Vari (Fig. 6G).

### *varicose *is required for normal wing and eye development

**Figure 7:**
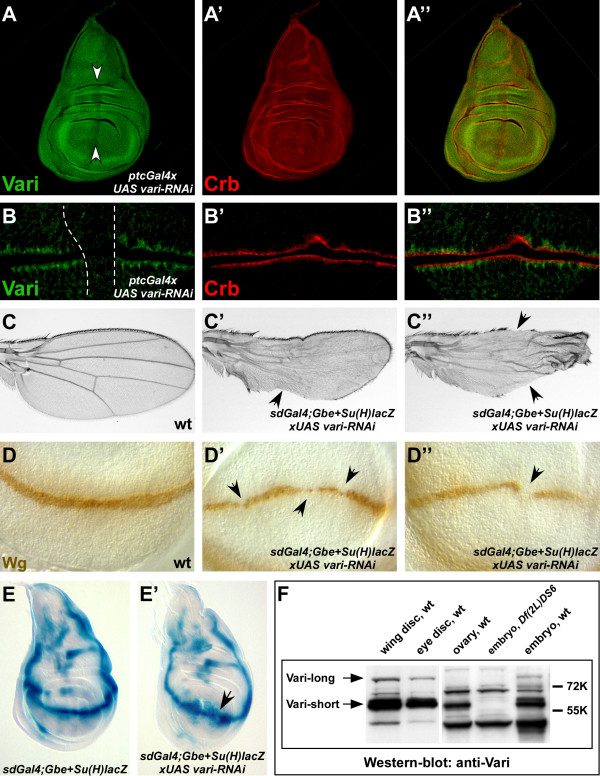
**RNAi-mediated knockdown of *vari *in the wing imaginal disc**. (A) Gal4/UAS-mediated overexpression of a *vari-RNAi *transgene along the antero-posterior boundary of a third instar wing imaginal disc with *ptcGal4 *significantly reduces the amount of Vari protein (white arrows). (B) Close-up view on imaginal disc cells from the wing imaginal disc shown in A. In the overexpression region (dotted white line in B) Vari (green) is no longer detectable at the SJ. Crb (red) still localises to the SAR, demonstrating that apico-basal polarity of the wing imaginal disc epithelial cells is unaffected. (C) Wing blades from adult wild-type flies (C) and flies in which the *vari-RNAi *transgene was expressed with *sdGal4; Gbe+Su(H)lacZ*. Overexpression of the transgene affects wing shape and margin formation to different degrees. Bristles and hairs are lost from the posterior and, more rarely, anterior wing margin, respectively (arrows in C', C"). The wings of adult *sdGal4; Gbe+Su(H)lacZ>UAS vari-RNAi *flies normally appear 'collapsed', but unfold again during the embedding procedure. (D) Anti-Wg antibody staining of *sdGal4; Gbe+Su(H)lacZ *(D) and *sdGal4; Gbe+Su(H)lacZ>UAS vari-RNAi *(D', D") third instar wing imaginal discs. In comparison to wild-type (D) *vari *knockdown leads to a lighter and discontinuous Wg expression at the prospective wing margin (arrows in D', D"). (E) X-Gal staining of *sdGal4; Gbe+Su(H)lacZ *(E) and *sdGal4; Gbe+Su(H)lacZ>UAS vari-RNAi *(E') third instar wing imaginal discs to visualize activity of the Notch signaling pathway. Notch activation at the prospective wing margin appears to be more diffuse and sometimes interrupted (arrow in E'). (F) Western blot analysis of protein lysates from wild-type wing and eye imaginal discs, wild-type ovaries as well as wild-type and *Df(2L)DS6 *embryos, probed with an anti-Vari antibody that recognises both Vari-long and Vari-short. Whereas in wing and eye imaginal discs both Vari-long and Vari-short can be detected, wild-type ovaries express only Vari-short. The protein amount loaded per lane equals 10 wing or eye imaginal discs, 1 ovary and 5 embryos, respectively. The lysates from wild-type and *Df(2L)DS6 *embryos serve as controls and the unspecific, cross-reacting band slightly below 72K as a loading control. In A, B, D and E anterior is left and dorsal is up. In C anterior is up.

**Figure 8:**
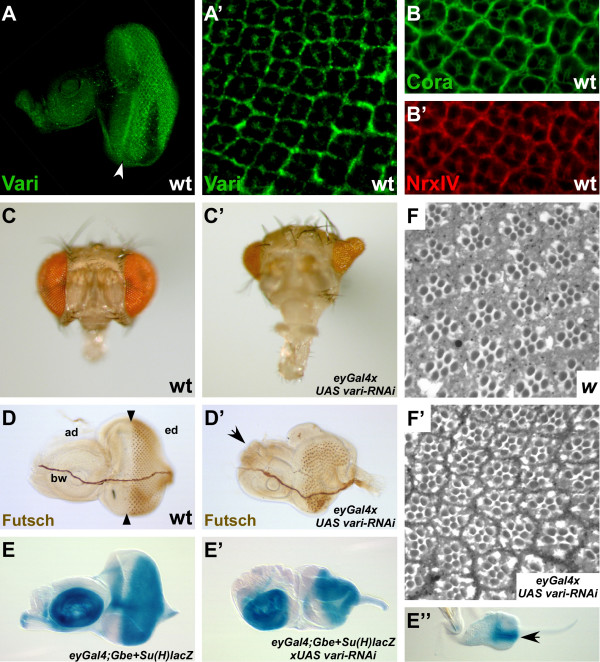
**Vari function in the developing eye**. (A, A') Third instar wild-type eye imaginal discs stained with anti-Vari (green). Vari is predominantly expressed behind the morphogenetic furrow (white arrow in A) in the developing ommatidia. The close-up view on ommatidia of the 4-cone cell stage (A') demonstrates that Vari localises to the lateral membranes of the photoreceptor and cone cells. (B, B') Close-up views of ommatidia, stained with antibodies against Cora (B, green) and NrxIV (B', red), respectively. Cora and NrxIV are expressed in the photoreceptor and cone cells. (C-C') Head of a wild-type fly (C) and a fly overexpressing *vari-RNAi *with *eyGal4 *(C'). Reduction of *vari *induces roughening, downsizing and malformation of the eye. (D-D') Eye imaginal discs of third instar wild-type larvae (D) and larvae overexpressing *vari-RNAi *with *eyGal4 *(D'), stained with anti-Futsch/22C10 antibody to mark the photoreceptor cells. Reduction of *vari *function (D') leads to aberrant folding of the eye imaginal disc and additional tissue formation at the antennal disc (arrow). Black arrowheads mark the position of the morphogenetic furrow in D. bw = Bolwig's nerve, ad = antennal disc, ed = eye disc. (E-E") X-Gal staining of *eyGal4; Gbe+Su(H)lacZ *(E) and *eyGal4; Gbe+Su(H)lacZ>UAS vari-RNAi *(E') third instar eye imaginal discs to visualize activity of the Notch signaling pathway. Despite morphological aberrations Notch activity does not seem to be strikingly affected by RNAi-mediated knockdown of *vari*. *eyGal4; Gbe+Su(H)lacZ>UAS vari-RNAi *eye imaginal discs from second instar larvae already display abnormal folding (E", arrow). The eye imaginal disc in E" is shown in a higher magnification than those in E and E'. (F, F') Semi-thin sections of eyes from wild-type flies (F) and flies overexpressing *vari-RNAi *with *eyGal4 *(F'). In ommatidia with reduced *vari *function (F') the seven photoreceptor cells visible do not exhibit major morphogenetic defects and are still organised in a trapezoid pattern as in wild-type (F). In A, D and E anterior is left.

The data show that *varicose *has an essential function during development of the embryo. Vari protein is also expressed at later stages, in the wing and the eye imaginal discs. In the wing imaginal disc, Vari is expressed throughout the disc epithelium, where it localises basal to Crumbs, which marks the apical pole (Fig. 7A,B). In the eye disc of third instar larvae, there is enriched expression of Vari behind the morphogenetic furrow (Fig. 8A). NrxIV and Coracle, two other components of the septate junction, exhibit the same expression pattern (8B, B') [27], suggesting that Vari is associated with SJ in differentiating ommatidia (Fig. 8A). In both the eye and the wing discs, both isoforms could be detected, while ovaries expressed only Vari-short, but not Vari-long (Fig. 7F).

To assess *vari *function in imaginal discs, we expressed double-stranded RNA (*vari-RNAi*) in a tissue-specific manner. Overexpression of *vari-RNAi *by means of *ptcGal4*, i. e. in a stripe of cells along the anterior-posterior compartment boundary of the wing imaginal disc, almost completely removed Vari protein from these cells (Fig. 7A,B), suggesting that *vari *function was strongly suppressed. Reduction of Vari had no effect on apical-basal polarity as revealed by the continuous apical localisation of Crumbs (Fig. 7B–B"). Use of *sdGal4 *to express *vari-RNAi *in the developing wing pouch from first/second larval instar onwards gave rise to adult flies with defective wings or pharate adults, depending on the strength of the different *vari-RNAi *effector-strains used. Hatched flies exhibited malformations of their wings to various degrees. Most prominent were gaps in the wing margin and defects in the unfolding of the wing (Fig. 7C–C").

*eyGal4*>UAS *vari-RNAi *animals, which expressed the *vari *dsRNA under the control of the *eyeless *promotor, i. e. in the eye imaginal discs from early stages onward as well as in a small stripe in the antennal disc [28], developed defects in the adult eye. These ranged from roughening of the eye to strong malformations and reduction in size (Fig. 8C,C'). In some cases, the eye tissue was replaced by head cuticle. In addition, some flies exhibited a partial or total loss of ocelli (data not shown). The defects in the eye could be traced back to the imaginal discs (Fig. 8D and 8E). The regular arrangement of the developing ommatidia was disturbed, and several additional folds and bulges were visible, both in the eye and the antennal part of the discs. However, patterning of cells within remaining ommatidia was unaffected, as revealed by histological sections of adult eyes lacking *vari *function. Photoreceptor cells were correctly arranged in a trapezoid pattern as in wild-type eyes (Fig 8F,F'). In order to determine the stage at which *vari *was required to support normal eye development, *vari-RNAi *was induced at different developmental stages making use of a temperature-sensitive inhibitor of Gal4, Gal80^ts ^[29]. Independent of the time point of induction of *vari-RNAi *expression (during embryogenesis, first or second larval instar), similar phenotypes with comparable penetrance and expressivity were obtained, although they were consistenly weaker than in flies that were permanently kept at 25°C (data not shown).

Some aspects of both the wing and the eye phenotypes obtained upon reduction of *vari *function by RNAi are reminiscent to phenotypes observed in animals in which Notch (N) signalling is reduced. *N *is haplo-insuffient, and removal of one copy of *N *or expression of a dominant-negative form of its ligand Serrate lead to notches in the wing [30,31]. Inactivation of the N pathway during early eye development results in reduction of eye size or complete lack of eyes [32-34]. To test whether *vari *affects the activity of the Notch pathway, we analysed the expression of a N activity reporter [35] in wing and eye discs expressing *vari-RNAi*. No major changes in the overall pattern of N activity were detected, neither in the wing (Fig. 7E–E') nor in the eye imaginal discs (Fig. 8E–E'), although the expression domains were often deranged/mislocalised due to the formation of extra folds and bulges, which were already visible in second instar larvae (Fig. 8E"). The only abnormality was observed at the prospective margin of wing imaginal discs, where the domain of N activation as well as expression of one of its target genes, *wingless*, were discontinuous in about a quarter of discs analysed (Fig. 7D–D" and 7E–E', arrows).

## Discussion

Here we demonstrate, that two out of three predicted Vari proteins are in fact expressed in the embryo. Both proteins are also present in imaginal discs of third instar larvae and heads of adult flies (data not shown), while in ovaries only Vari-short could be detected. In the embryo, the two isoforms are differentially expressed, the smaller one being expressed earlier and much more abundant than the larger form. Localised Varicose protein is detected even later, after stage 12. This, together with the analysis of *vari *transcripts and proteins in the different mutants suggests that there are several levels of gene expression regulation. For example, in *vari*^03953*b *^the longer transcript is highly upregulated, which is not reflected at the protein level. Here, the near absence of the small isoform seems to have an effect on the synthesis and/or stability of the longer isoform. In *vari*^38*EFa*2 ^mutant embryos the truncated *vari-long *transcript is very abundant, which is not associated with more (truncated) Vari-long protein, pointing to less efficient translation an/or reduced stability of the mutant protein. In contrast, the truncated *vari-short *transcript is strongly reduced in abundance, which is not paralleled by a reduction in protein levels. In embryos mutant for this allele, properly localised protein can be detected, though in lower amounts (data not shown). Since the Vari antibody used does not allow discriminating between the two proteins, we cannot determine whether both isoforms are correctly localised. Although in *vari*^*MD*109^, *vari*^327 ^or *vari*^*R*979 ^one or both isoforms are synthesised in normal size and amount, in no case these proteins are correctly localised at the membrane [this work and [13]].

Although not predicted by commonly used domain prediction programs, sequence comparison of Vari-long with its closest vertebrate orthologues, MPP2_b, MPP6_c, and other related MAGUKs, makes the presence of a second, more divergent N-terminal L27 domain in the longer Vari isoform very likely. This situation is similar to the MAGUK Stardust, the L27N domain of which also fits less to the canonical sequence [5,36]. The expression of two isoforms of a MAGUK protein, which differ by the presence or absence of the L27 domain(s) is not unique. For example, one close *vari *orthologue, human *MPP6*, also encodes two isoforms, one of which, MPP6_a, lacks the two L27 domains (Fig. 1B). The human postsynaptic density (PSD)-95 protein and *Drosophila *Discs large also come in two variants, one with and one without a L27 domain, respectively [4,37]. In the case of Discs large, the DlgA isoform is predominantly expressed in the embryo and Dlg-S97 in the adult brain, but both isoforms are co-expressed in the larval NMJ [38]. In the case of Vari, both isoforms are expressed in embryonic epithelia. Since the antibody used here recognises an epitope common to both Vari isoforms, we cannot completely rule out the possibility that different embryonic epithelia express different Vari isoforms, although this seems unlikely due to the interdependence between Vari-long and Vari-short. Our data further suggest that they are localised at the septate junction, since both co-immunoprecipitate with NrxIV-GFP, which is localised in the septate junction. Targeting and/or stabilisation of Neurexin IV are probably mediated by direct interaction between the PDZ domain of Vari and the C-terminal amino acids of Neurexin IV. However, other partners of Vari, particularly those interacting with the L27 domains, are still elusive. Similar as human VAM-1/MPP6_c, which binds human Veli-1 in vitro [39], the L27 domain of *Drosophila *Vari can interact with the L27 domain of *D*Lin-7 in vitro. The different localisation of the two proteins, at least in epithelia of the *Drosophila *embryo, however, makes their in vivo interaction in these cells very unlikely.

What could be the significance of expressing two Vari isoforms? L27 domains can mediate the targeting of the respective protein to a particular membrane compartment, such as the synapse or the adherens junction, or stabilise interacting proteins by directly binding to the L27 domain of the respective partner [37,40]. On the other hand, MAGUK proteins without L27 domains can efficiently be brought to their proper site, using other targeting mechanisms, for example palmitoylation [41]. This raises the question as to i) whether the two Vari proteins rely on different mechanisms for targeting to the septate junctions and ii) whether the two Vari isoforms have specific functions in the *Drosophila *embryo. Using Gal4-mediated overexpression, either of them is capable to rescue *vari*^*F*033 ^mutant embryos to viability. This allele has been classified as a null allele, based on its genetic behaviour [13] and the complete and nearly complete lack of Vari-long and Vari-short, respectively (this work). The rescuing capability of Vari-short indicates that the L27 domains are not essential for viability, but does not exclude any non-essential function, in the embryo or at later stages. Strikingly, the hypomorphic allele *vari*^38*EFa*2^, which still expresses the short Vari isoform, but a modified Vari-long protein, gives rise to weak, but viable and fertile adults. The deletion in this allele removes 51 amino acids in the N-terminus, which affects both L27 domains. The fact that the escapers do not exhibit any mutant eye or wing phenotype similar to that obtained upon RNAi-mediated knockdown of *vari *in imaginal discs suggests a more supportive function for the larger isoform. The predominant role of Vari-short is further highlighted by the fact that *vari*^*MD*109^, in which Vari-short is absent, is lethal, despite expression of normal Vari-long proteins. Hence, physiological amounts of only Vari-long are not sufficient for viability, but excess levels and/or earlier expression (using *da*G32/*daughterlessGal4*) can restore viability in the absence of Vari-short.

Mutational analysis of *vari*^327 ^uncovered a five amino acid deletion in the SH3-domain of Vari, which almost completely removes one of the four core β-strands present in all SH-3 domains, thus completely abolishing Vari function. Although both Vari isoforms are expressed in *vari*^327 ^in wild-type amounts, this allele is a functional null and no localised protein could be detected. The fact that the mutant proteins are not localised suggests that either the SH3 domain is necessary for targeting, or that the overall structure of the protein is affected, preventing proper localisation. Structural and functional analysis of other MAGUKs, e. g. PSD-95 or hCASK, point to either intra- or intermolecular interactions between the SH3 and the GUK domain [42,43]. In the MAGUK PSD-93, binding of a ligand to the PDZ domain releases intramolecular inhibition of the GUK domain by the SH3 domain [44].

A strong reduction of *varicose *function by RNAi in postembryonic stages also affects the normal development of eyes and wings. It is interesting, however, to note that the consequences of RNAi-mediated knockdown of *vari *in wing and eye imaginal discs seem to be different. Reduced *vari *function in wing imaginal discs attenuates N signaling, as revealed by lowered activity of a N reporter gene construct and disrupted expression of the N target gene *wingless *at the prospective margin of wing imaginal discs. In wild-type wing imaginal discs *N *is activated on both sides of the dorsal/ventral compartment boundary by its two ligands, dorsally expressed Serrate and ventrally expressed Delta. N activity in the wing margin activates downstream genes, like *cut *or *wingless*, which are involved in the regulation of growth and patterning [reviewed in [45]]. Reduction of N activity results in the formation of notches in the margin, as observed here. In contrast, the N reporter seems to be normally activated in eye imaginal discs upon *vari *RNAi induction. Eye imaginal discs with reduced *vari *function display abnormal folding and adult eyes are smaller and misshapen. Additionally, ocelli and bristles are sometimes replaced by bare head cuticle. This phenotype is reminiscent of eye phenotypes observed in certain allelic combinations of *coracle*, which exhibit roughening of the eyes due to abnormally spaced ommatidia, but without affecting the patterning of the photoreceptor cells, and often lack ocelli and bristles. In addition, some *coracle *mutations suppress the effects of hypermorphic mutations in the EGF-receptor [18].

This suggests that SJs are differentially required for normal signalling at various developmental stages, but may affect different signalling pathways in a tissue dependent way. Given that SJs are required throughout imaginal disc development as suggested by our results, their lack may affect various signalling pathways, which are spatially and developmentally regulated and integrated as shown for the EGF-receptor and Notch pathway [46]. So far, nothing is known how SJs may affect signalling. They could be involved in the correct localisation of receptors, membrane-bound ligands and/or components involved in signal transduction. The stage and tissue-dependent differential contribution of various signalling pathways may explain the different phenotypes obtained upon RNAi-mediated *vari *knockdown in eye and wing discs.

Septate junctions in larval eye imaginal discs have been well documented before [47], but their exact role during postembryonic development is largely unknown. *NrxIV *has recently been shown to be required for septate junction formation between and among the cone and pigment cells and for ommatidial morphology and integrity [27]. Some of the phenotypes observed in *NrxIV *mutant clones in the developing eye, which are reminiscent to those obtained by *eyGal4*-mediated overexpression of *vari-RNAi*, have been interpreted as the result of compromised adhesion. Based on the molecular and genetic interaction between NrxIV and Vari, it is tempting to speculate that *vari *has a comparable role during eye development. In addition, during morphogenetic movements, cell divisions and cell rearrangements, SJs have to be redistributed [48]. Loss of *vari *may thus interfere with these processes, which could explain the abnormal folding of *eyGal4>UAS vari-RNAi *eye imaginal discs.

Our data suggest that the final *vari *mutant phenotype is the consequence of compromised SJ function at different stages of development, which, in turn, may affect several cell-cell signalling and adhesion processes. A detailed dissection of the complexity of the mutant phenotype is beyond the scope of this manuscript. In the future it provides, however, a well-suited system to study the postembryonic function of SJs.

## Conclusion

Implications from our findings are threefold. i) *varicose *is required for septate junction development in *Drosophila *embryos. ii) Expression of two Varicose isoforms in embryonic epithelia and imaginal discs suggests that the composition of Varicose-mediated protein scaffolds at septate junctions is dynamic. iii) *varicose *is required for normal wing and eye development.

## Methods

### Fly stocks

The following fly stocks were used: wild-type (Oregon R), *Gbe+Su(H)lacZ *[35], *NrxIV-GFP/TM6B *(Nadine Muschalik and E. Knust, unpublished), *P(EP)Nrx-IV*^*EP*604 ^(Bloomington/17185), *Df(2L)DS6 *(Bloomington/2386), *Df(3L)BK9 *(Bloomington/2991), *P(XP)d10880 *[49], *vari*^*MD*109 ^(see below), *vari*^03953*b *^[26], *vari*^38*EFa*2 ^[50], *vari*^*F*033 ^[49], *vari*^327^, *vari*^*R*979^, *vari*^*R*3^, *UAS vari-short *[13], *UAS Flag-vari-long *(see below), *UAS vari-RNAi *(see below), *UAS eGFP-dlgA *[51], *UAS DLin-7-EGFP *(A. Bachmann and E. Knust, unpublished). UAS constructs were activated using *sdGal4 *[52], *enGal4 *[53], *da*G32 [54], *eyGal4 *(Bloomington/5535), *ptcGal4 *[55] or *btlGal4*, *UAS CD8-GFP *(kindly provided by D. Rosin) for overexpression in a wild-type genetic background and rescue experiments, respectively. *eyGal4; Gal80ts/TM6 *flies (kindly provided by C. Klämbt) were used for temperature-shift experiments. A *CyO-twi-GFP *balancer chromosome (Bloomington/6662) was used for selecting homozygous *vari*-mutant embryos.

### Generation of the *vari*^*MD109 *^mutant

P-element line *P(XP)d10880 *is derived from the Exelixis insertion collection [49]. The P-element is inserted in front of the *vari-short *specific exon 3a at position 2L: 23011544 (release: r5.2). Imprecise excision of *P(XP)d10880 *yielded the mutant allele *vari*^*MD*109^, which carries a 1103bp deletion that removes exon 3a and part of the following intron, but still contains 464bp of the *P(XP) *vector sequence.

### Sequencing of the *vari^327 ^*allele and BLAST analysis

Genomic DNA from GFP-selected *vari*^327 ^embryos was isolated as follows: 50 embryos were homogenized in 50 μl squishing buffer (10 mM Tris-HCl pH 8, 1 mM EDTA, 25 mM NaCl, 200 μg/ml Proteinase K) and incubated for 30 min at room temperature. Proteinase K was inactivated by heating to 90°C for 2 min. 5 μl genomic template DNA were used for PCR amplification with the following primers: vari-PDZ/SH3-5 = GGA CGT CGA CAA CAC CAA GAT GC and vari-short-3 (see below). The purified PCR product was sequenced using the vari-PDZ/SH3-3 primer as sequencing primer (Eurofins MWG).

Sequence comparison was kindly performed by Bianca Habermann, MPI Dresden, using reciprocal BLASTs [56] to find orthologues/paralogues. The domain search with the vertebrate and honey bee MAGUKs was done using SMART [57]. Sequences were aligned using ClustalW [58] with manual refinement.

### Transgenic constructs and germline transformation

An amino-terminally Flag-tagged version of Vari-long was generated by introducing a PCR-amplified *vari-long *cDNA (BDGP DGC clone RE31492 as template; primers: CG9326-Not-5 = CCT TCA AAG CGG CCG CGT GGA GCG; CG9326-Asp-3 = GCT TCA GGT ACC GGA ATT ATC CC) into the pUAST-Flag vector [5]. The construct for *vari*-specific DNA-mediated RNA interference was generated by introducing a PCR-amplified *vari *fragment covering part of the SH3 and the complete GUK domain (Pro^325 ^to Trp^629^; BDGP DGC clone RE31492 as template; primers: vari-RNAi-5 = CAT CAA CGT AAA GGA TCC CAA CTG G; vari-RNAi-3 = GTT GAC AGG TAC CCA CTG CTC CTC G) into pHibs followed by cloning into pUAST as described [59]. Transgenic flies were generated by P-element-mediated germline transformation following standard procedures [60].

### Temperature shift experiments

Eggs of the genotype *eyGal4/+; Gal80ts/UAS vari-RNAi *were collected from a synchronous culture for 1 hour. Egg collections were reared at 18°C except for a single shift to 29°C in a 12 hour time window. The single 12 hour heat-shock of each sample was performed during different periods of development spanning from zero hours after egg laying to the late second larval instar. After the 12 hour exposure to 29°C cultures were returned to 18°C for the remaining development until adult flies emerged.

### RT-PCR, in situ hybridisation and northern blotting

RT-PCR was performed on total RNA isolated from 100 GFP-selected *vari *and *vari/CyO-twi-GFP *embryos, respectively, using the NucleoSpin RNA II kit from Macherey-Nagel. 250 ng of total RNA were used as template for each RT-PCR reaction (OneStep RT-PCR Kit, Qiagen). The *vari-long *and *vari-short *transcripts were detected using the following primer pairs: *vari-long*: vari-long-5 = CTG TCC TCC GGG TCG ACT TCA TTC and vari-long-3 = GCC ATT CTG TGG TAC CTG ACC CC; *vari-short*: vari-short-5 = TCA GTT AGT TGC GAA CAG TGC TAA and vari-short-3 = CTC GCA AGC GGC CGC AGA TC. As an internal loading control transcripts from the *rp49 *gene were detected using a rp49-5 primer = AGA TCG TGA AGA AGC GCA CC and a rp49-3 primer = CGA TCC GTA ACC GAT GTT GG.

Digoxygenin-labelled RNA sense and antisense probes were generated based on the full-length *vari *cDNA RE31492 (BDGP DGC) using the DIG RNA labeling Kit (Roche). In situ hybridisation and northern blotting were performed following standard procedures.

### Antibodies, immunofluorescence, X-Gal staining and electron microscopy

Antisera against Vari were obtained by repetitive immunization of a rabbit and a rat with an affinity-purified GST-Vari fusion protein (covering the PDZ and SH3 domains, Asn^165 ^to Ser^384^; Eurogentec). For immunohistochemistry, embryos and imaginal discs were fixed and stained by standard protocols. Rabbit anti-Vari was used at 1:1.000, rat anti-Vari at 1:100. Other primary antibodies used were mouse anti-Wg^4D4 ^(1:50), mouse anti-Futsch^22c10 ^(1:50), mouse anti-Dlg^4F3 ^(1:100), mouse anti-FasIII^7G10 ^(1:5) and mouse anti-Arm^N2-7A1 ^(1:25) (Developmental Studies Hybridoma Bank), rat anti-*D*Lin-7 (1:100) (A. Bachmann and E. Knust, unpublished), rat anti-Crb^2.8 ^(1:100) (E. Theilenberg and E. Knust, unpublished), rabbit anti-NrxIV (1:500) [14] and guinea pig anti-Cora (1:2.000) [61]. Fluorescence-labeled secondary antibodies purchased from Jackson ImmunoResearch Europe Ltd. or Molecular Probes (Invitrogen) were applied at a 1:200 dilution. Confocal imaging was performed on a Leica TCS NT confocal microscope. All images were processed and mounted using Adobe Photoshop 7.0 and Deneba Canvas 9.0.

Staining for *lacZ *activity in the imaginal discs was performed as described [31].

Semi-thin sections of adult eyes were prepared as described [62]. Transmission electron microscopy of GFP-selected *vari *and *vari/CyO-twi-GFP *embryos, respectively, was essentially performed according to Tepass and Hartenstein [9].

### Pull-down assay, immunoprecipitation and western blotting

*GST-NrxIV*^*intra *^and *GST-NrxIV*^*intra*-*stop *^constructs encode the intracellular domain of NrxIV, fused to the C-terminus of GST. They were cloned by introducing *NrxIV *PCR products from genomic DNA into pGex-4T1 (GE Healthcare): *GST-NrxIV*^*intra *^(primers: NrxIV^intra^-5 = GCT GCT GAT CCT TGA ATT CTT CCT TAT C and NrxIV^intra^-3 = GCG TCT CGA GCC TTT TCC GTG), *GST-NrxIV*^*intra*-*stop *^(primers: NrxIV^intra^-5 = see above and NrxIV^intra-stop^-3 = CGG TTT AGC TCG AGA TCT CTT ATC GC). His-tagged *vari*^*PDZ *^was constructed as follows: the coding region for the *vari *PDZ domain was amplified from BDGP DGC clone RE31492 by the use of primers QE-vari-PDZ-5 = AAC ACC AAG AGG CCT GTG GAG ACC and QE-vari-PDZ-3 = GCC ATT CTG TGG TAC CTG ACC CC, followed by cloning into pQE-30 Xa (Qiagen).

*GST-DLin-7 *is described elsewhere [5]. *GST-DLin-7-PDZ *and *GST-DLin-7-L27 *were constructed by introducing the following PCR products from a *D*Lin-7 cDNA into pGex-4T1 (GE Healthcare): *GST-DLin-7-PDZ *primers: *D*Lin-7-PDZ-5 = CAC TGT GGC AGA ATT CGC AGC and *D*Lin-7-PDZ-3 = GAA CTA GTC GAC CTG TAT GGT C; *GST-DLin-7-L27 *primers: *D*Lin-7-L27-5 = TTG AAC AGC GCG GCC GCC GAT AAC and *D*Lin-7-L27-3 = GGG CAG GTC GAC GAC TCT GGG. His-tagged *vari*^*L*27 ^was constructed as follows: the coding region for the *vari *N-terminus including the L27 domains was amplified from BDGP DGC clone RE31492 by the use of primers QE-vari-L27-5 = CAA AAT GGT TAG GCC TAG CGC G and QE-vari-L27-3 = GGT GAA CAG GTA CCC TCC CGT TT, followed by cloning into pQE-30 Xa (Qiagen).

Induction and purification of bacterially-expressed fusion proteins was done according to the manufacturers instructions.

The GST-pull-down assay was done essentially as described [5]. Eluted labelled proteins were analyzed by SDS-PAGE and detected with a mouse anti-His antibody (1:2.500, Qiagen). Immunoprecipitation experiments from embryos were performed as described [63] with the following modifications: *NrxIV-GFP/TM6B *embryos (> 8 hours old) were lysated in CHAPS lysis buffer (20 mM Tris-HCl pH8, 150 mM NaCl, 10% glycerol, 2 mM EDTA, 10 mM CHAPS and protease inhibitors: 1 μM Pefabloc, 5 μM Leupeptin, 1 μM Pepstatin, 0.3 μM Aprotinin). Immunoprecipitation was performed with 10 μl of mouse anti-GFP antibody (Roche) and control antibody (2 μl mouse anti-β-gal^40-1a^ antibody, Developmental Studies Hybridoma Bank), respectively. Varicose was detected using the rabbit anti-Vari antibody at 1:2.000.

## Authors' contributions

AB generated constructs and transgenic flies, carried out RT-PCR, in situ hybridisation, X-Gal and antibody stainings, and localisation studies. Additionally, AB performed the Western blot analysis, interaction studies and participated in experimental design, data analysis and writing of the manuscript. MD did the northern blot, generated constructs and transgenic flies, created the *vari*^*MD*109 ^mutant, did antibody stainings and localisation studies. FG carried out electron microscopy and semi-thin sections. EK was responsible for overall experimental design, analysis and interpretation of data and writing of the manuscript. All authors read and approved the final manuscript.

## Supplementary Material

Additional file 1 Sequence alignment of Vari with its closest orthologues. *Am: Apis mellifera, Xt: Xenopus tropicalis, Mm: Mus musculus, Dr: Danio rerio*. The domain boundaries are according to murine MAGUK2. Click here for file
