# Supplementary material for: On the role of the MAGUK proteins encoded by Drosophila varicose during embryonic and postembryonic development
Source: BMC Dev Biol. 2008 May 18;8:55. doi: 10.1186/1471-213X-8-55 (PMC2414870; doi:10.1186/1471-213X-8-55)
Supplement: Additional file 1 — Sequence alignment of Vari with its closest orthologues. Am: Apis mellifera, Xt: Xenopus tropicalis, Mm: Mus musculus, Dr: Danio rerio. The domain boundaries are according to murine MAGUK2. [file 1471-213X-8-55-S1.pdf]

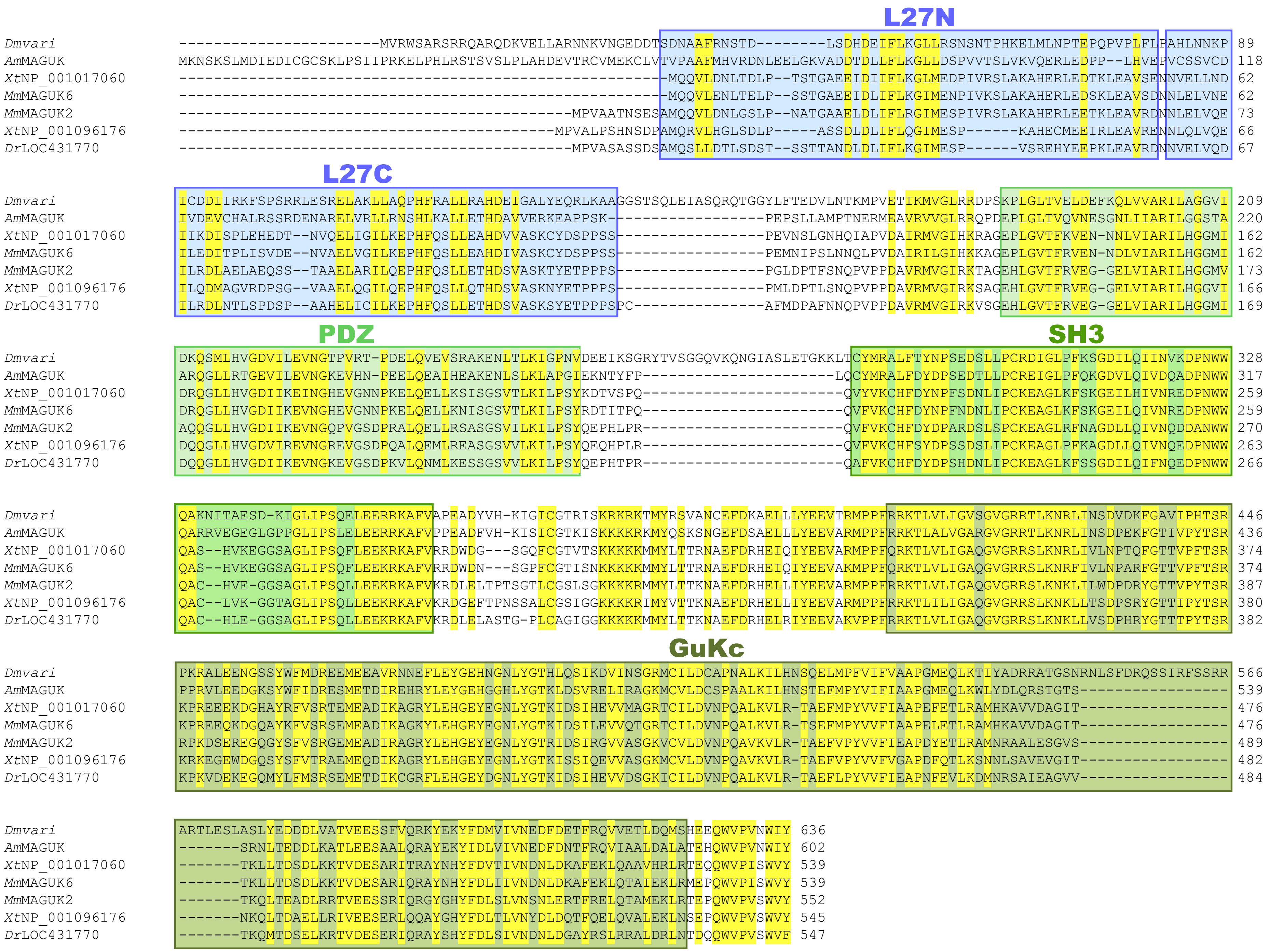

L27N
L27C
PDZ
SH3
GuKc
Dmvari
AmMAGUK
XtNP_001017060
MmMAGUK6
MmMAGUK2
XtNP_001096176
DrLOC431770
